# Improving Dental Implant Outcomes: CNN-Based System Accurately Measures Degree of Peri-Implantitis Damage on Periapical Film

**DOI:** 10.3390/bioengineering10060640

**Published:** 2023-05-25

**Authors:** Yi-Chieh Chen, Ming-Yi Chen, Tsung-Yi Chen, Mei-Ling Chan, Ya-Yun Huang, Yu-Lin Liu, Pei-Ting Lee, Guan-Jhih Lin, Tai-Feng Li, Chiung-An Chen, Shih-Lun Chen, Kuo-Chen Li, Patricia Angela R. Abu

**Affiliations:** 1 Department of General Dentistry, Keelung Chang Gung Memorial Hospital, Keelung City 204201, Taiwan; 2 Department of General Dentistry, Chang Gung Memorial Hospital, Taoyuan City 33305, Taiwan; 3 Department of Electronic Engineering, Chung Yuan Christian University, Taoyuan City 32023, Taiwan; g10976016@cycu.edu.tw (T.-Y.C.);; 4 School of Physical Educational College, Jiaying University, Meizhou 514000, China; 5 Department of Electrical Engineering, Ming Chi University of Technology, New Taipei City 243303, Taiwan; joannechen@mail.mcut.edu.tw; 6 Department of Information Management, Chung Yuan Christian University, Taoyuan City 320317, Taiwan; kuochen@cycu.edu.tw; 7 Ateneo Laboratory for Intelligent Visual Environments, Department of Information Systems and Computer Science, Ateneo de Manila University, Quezon City 1108, Philippines; pabu@ateneo.edu

**Keywords:** peri-implantitis, periodontitis, periapical radiograph, deep learning, neural networks, image enhancement

## Abstract

As the popularity of dental implants continues to grow at a rate of about 14% per year, so do the risks associated with the procedure. Complications such as sinusitis and nerve damage are not uncommon, and inadequate cleaning can lead to peri-implantitis around the implant, jeopardizing its stability and potentially necessitating retreatment. To address this issue, this research proposes a new system for evaluating the degree of periodontal damage around implants using Periapical film (PA). The system utilizes two Convolutional Neural Networks (CNN) models to accurately detect the location of the implant and assess the extent of damage caused by peri-implantitis. One of the CNN models is designed to determine the location of the implant in the PA with an accuracy of up to 89.31%, while the other model is responsible for assessing the degree of Peri-implantitis damage around the implant, achieving an accuracy of 90.45%. The system combines image cropping based on position information obtained from the first CNN with image enhancement techniques such as Histogram Equalization and Adaptive Histogram Equalization (AHE) to improve the visibility of the implant and gums. The result is a more accurate assessment of whether peri-implantitis has eroded to the first thread, a critical indicator of implant stability. To ensure the ethical and regulatory standards of our research, this proposal has been certified by the Institutional Review Board (IRB) under number 202102023B0C503. With no existing technology to evaluate Peri-implantitis damage around dental implants, this CNN-based system has the potential to revolutionize implant dentistry and improve patient outcomes.

## 1. Introduction

In recent decades, dental implant technology has gained popularity, boasting a success rate of over 90% for artificial dental implant surgery [1]. The human mouth contains 32 permanent teeth, each with an interlocking function. Missing teeth lead to a cascade of oral health issues, causing more significant long-term damage than adjacent natural teeth [2]. Failure to address a missing tooth can lead to tooth decay and peri-implantitis, impairing the original function of the mouth. In more severe cases, adjacent teeth can shift, bone shrinkage can occur, and bite and temporomandibular joint disorder (TMD) can develop [3,4]. Symptoms associated with TMD include Temporomandibular Joint (TMJ) pain, chewing pain, pain around the ear, and facial asymmetry due to uneven force application [5,6]. According to the American Dental Association, around 5 million dental implants are annually implanted in the U.S., and the worldwide market for dental implants is projected to reach USD 4.6 billion by 2022 [7]. Today, dental implants are a common dental procedure, involving the surgical implantation of a titanium root into the alveolar bone where a tooth is missing [8]. After sterile treatment and a secure bond between the root and tissue, an artificial crown is placed to replace the missing tooth [9]. The structure of the implant is similar to that of a natural tooth and will not cause any foreign body sensations when biting [10].

The use of artificial intelligence (AI) has become prevalent across various fields due to technological advancements. In recent years, the integration of AI and medicine has emerged in areas such as Cardiology [11], Pulmonary Medicine [12], and Neurology [13]. Artificial intelligence can help doctors to consolidate data and provide diagnostic methods. It also brings medical resources to rural areas to improve the quality of medical care around the world, which shows that artificial intelligence is extremely helpful to society [14]. The combination of Convolutional Neural Networks (CNN) and dentistry has resulted in a wealth of information. Research in AI has displayed promising results in utilizing the three common X-ray film types used in routine dental exams, including Panoramic radiographs, Periapical films, and Bite-Wing films. In the realm of dental radiology research, two primary areas of focus are tooth localization and identification of disease symptoms. Image enhancement techniques have been proposed to increase the accuracy of cutting and positioning of individual teeth. For instance, some studies have utilized a polynomial function to connect gap chains into a smooth curve, resulting in a 4% improvement and 93.28% accuracy rate [15]. Additionally, Gaussian filtering and edge detection technology have been proposed to enhance the visibility of tooth gaps and facilitate the cutting and positioning of individual teeth [16]. Filters have been helpful in reducing the impact of point creation on cutting technology and recognition in PA [17]. Furthermore, adaptive thresholds have been suggested to improve the application of cropping technology in dental radiology research [18]. Regarding the identification of disease symptoms, the backpropagation neural network has been used to diagnose dental caries with an accuracy rate of 94.1% [19]. Tooth detection and classification have been carried out on panoramic radiographs by training and classifying tooth types into four groups using a quadruple cross-validation method with 93.2% accuracy. Dental status has also been classified into three groups with an accuracy of 98.0% [20]. These findings demonstrate the enormous potential of AI in the dental field, with the ability to provide accurate diagnosis and improve patient care.

The dental implant surgery carries potential complications such as sinus perforation or jaw paralysis due to its location in nerve-ridden gums [21], making focus and attention crucial to avoid medical disputes. Currently, the objective of research in this area focuses on two areas: inspection and pre-operative analysis, thus reducing clinic time for dentists and enabling them to focus on treatment and technique. For example, CNN technology has been used for whole oral cavity analysis and inspection of periapical radiographs during the inspection stage [22,23]. Other studies have proposed an automatic synchronous detection system for two-dimensional grayscale cone beam computed tomography (CBCT) images of alveolar bone (AB) and the mandibular canal (MC) for preoperative treatment planning [24]. Additionally, pain and discomfort during the operation can affect its smoothness, and some research has proposed evaluating and predicting pain [25]. However, there is a limited amount of research conducted on postoperative analysis. Insufficient cleaning by the patient may result in peri-implantitis [26,27], wherein bacteria can gradually erode the tissues surrounding the implant, leading to bone and flesh loss. As a result, the implant may lose support and become loose or dislodged. In view of this, the aim of this study is to assess the extent of periodontal damage surrounding implants and provide accurate and objective evaluation results for postoperative follow-up examinations. The study aims to decrease the workload of dentists, protect the rights and interests of patients, and prevent potential medical disputes. This proposal provides three contributions and innovations:

1. The YOLOv2 model is trained using the manually created ROI database provided by the dentist to detect the implant position and return data for individual implant thread cropping;
2. Histogram equalization, overlapping techniques, and adaptive histogram equalization are employed to enhance the boundary lines. Additionally, the gingival area is colored orange, while the threaded area is green, thereby improving subsequent CNN judgment;
3. The study trains preprocessed data in a CNN model to detect damages, utilizing the AlexNet algorithm, achieving a final accuracy rate of 90.4%. Additionally, this research presents the first medical assistance system for automated thread analysis of implants.

The structure of this paper is as follows: Section 2 introduces the use of deep learning models for implant location labeling, cropping, and anterior processing, and finally, the use of CNN to build a model for arguing whether there is damage; Section 3 mainly integrates the methods used and the research results; Section 4 discusses the experimental results; and Section 5 describes the conclusion and future prospects.

## 2. Materials and Methods

**Figure 1:**
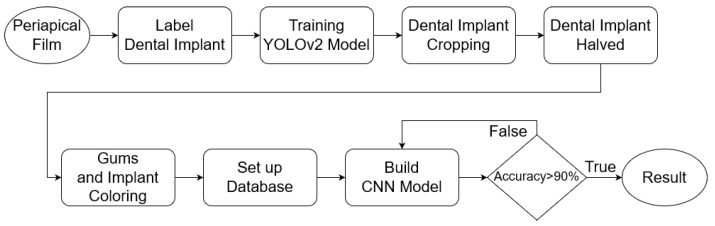
The flowchart of this research.

The database used in this research is collected from relevant cases diagnosed by professional dentists. It can be roughly divided into three parts: implant cropping, image preprocessing, and implant classification. The damages of dental implants are determined by the M(mesial) and the D(distal). Therefore, the step of implant cropping will be divided into cutting out single implants from one to multiple implants in the PA. This part will use a deep learning model to label the implant position and then separate it into M and D by using the linear regression algorithm. Although both implant cropping and implant classification require machine learning, the training methods are very different. Not only are different models used but also different types of databases are introduced. The implant cropping is trained using a manually selected ROI database, while the implant classification is trained using preprocessed images. The major problem encountered in this research is that the validation set cannot converge when the cropped implant images are directly fed into the model for training, which leads to overfitting. To solve this problem the research colors different parts of the implant image, adding reference lines and adjusting the parameters of the CNN model. The flowchart of this research is shown in Figure 1.

### 2.1. Image Cropping

To enable the CNN model to focus specifically on identifying destruction of dental implants on the mesial and distal sides, the PA image needs to be cropped to a single implant. Manual cropping is a time-consuming process. This study utilizes YOLOv2 to detect the position of the implant. Using the position information returned by YOLOv2, the implant can be cropped efficiently. Next, a linear regression algorithm is used to find the central cutting line of the implant. The output image is then named and classified by comparing it with the diagnosis results provided by the hospital, creating a database for the CNN model. To prepare the data for further analysis, image preprocessing is then performed.

#### 2.1.1. Label Dental Implant

**Figure 2:**
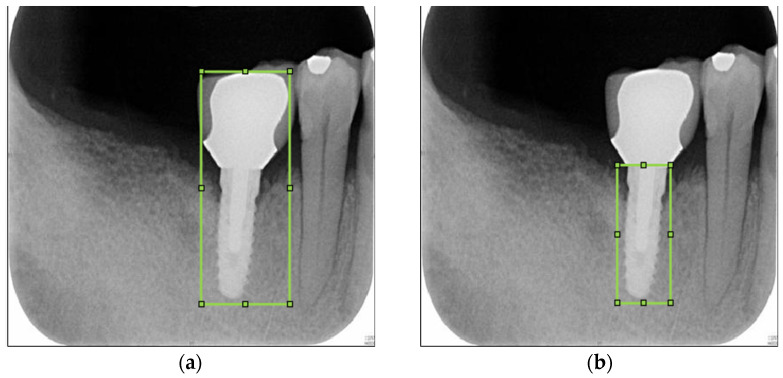
Illustrating the marking of the Region of Interest (ROI) in this study. (**a**) The dental implant is depicted, including the area above the screw thread. (**b**) Only dental implants with screw threads are included in the ROI.

The key issue in this step is to determine the Region of Interest (ROI) for training the object detection model. If the ROI encompasses the entire implant, the damage feature of the screw thread may not be classified accurately. This is because the area above the screw thread occupies most of the picture as depicted in Figure 2a, which can also make subsequent cropping steps challenging. Labeling only the screw thread, on the other hand, will not affect the determination process. Hence, the research sets the ROI to the thread instead of the implant body as shown in Figure 2b, to preserve the damage features of the screw thread as much as possible. Additionally, the damage detection also requires the gingival features surrounding the screw thread. Therefore, in the subsequent step of cropping the screw thread, the ROI returns the position that expands horizontally by several pixels to preserve these features for the next step.

#### 2.1.2. YOLOv2 Model

The main purpose of training the object detection model in this study is to improve operational efficiency and reduce the time required for manual image cropping. Therefore, this study uses specific instruments to achieve the best training effect, including hardware equipment, as listed in Table 1; software, i.e., YOLOv2 layer structure model, as listed in Table 2; and training parameter settings, as listed in Table 3.

To train the YOLOv2 model to label the position of an implant, this research manually labels a total of 211 photos with 147 used for training and 64 for testing. The remaining 173 images are labeled directly by the YOLOv2 model as indicated in Table 4. Ultimately, the position of an implant is exported to the next step while the confusion matrix is calculated using the results of the following step. By employing this approach, the research can reduce the time required for manual image cropping and achieve accurate labeling of implant positions. This is accomplished by training the YOLOv2 model to identify the position of the implant within the image. The ability of the YOLOv2 model to identify the location of the implant quickly and accurately allows for efficient and accurate cropping of the image, therefore reducing the amount of time required for this process. To ensure the accuracy of the YOLOv2 model, manually labeling a significant portion of the images used for training was conducted in this research. This manual labeling allowed for the evaluation of the performance of the model and made any necessary adjustments to improve its accuracy. The remaining images were labeled by the YOLOv2 model to further improve its accuracy.

In conclusion, the object detection of the model training is critical to reducing the time required for manual image cropping in this research. The use of hardware and software configurations was optimized for this purpose along with the manual labeling of images, thus allowing the YOLOv2 model to accurately identify implant positions in the image. By doing so, this proposed study can achieve efficient and accurate image cropping, therefore reducing the amount of time required for this process.

##### Optimizer

Optimizers play a crucial role in machine learning by helping to minimize the loss function. The choice of optimizer depends on the specific network and the problem at hand. In MATLAB, there are several options for optimizers, including Sgdm, RMSProp, and Adam.

The Sgdm optimizer is a variant of stochastic gradient descent with momentum, which uses the gradients of the current mini-batch and the previous mini-batch to update the model parameters. It has been shown to be effective in improving convergence speed and reducing the likelihood of becoming stuck in local optima. RMSProp optimizer, on the other hand, adjusts the learning rate adaptively for each parameter based on the average of the squares of the gradients. It is known to be useful for training recurrent neural networks. Adam optimizer is another popular algorithm that combines the ideas of momentum and adaptive learning rates. It has been shown to be effective in training large-scale deep learning models.

For this research, the Sgdm optimizer was chosen for the YOLOv2 network. The reason for this choice may be related to its effectiveness in improving convergence speed, reducing the likelihood of becoming stuck in local optima, and its ability to handle large datasets. Ultimately, the choice of optimizer depends on the specific problem being addressed and the characteristics of the data.

##### Initial Learning Rate

The initial learning rate is a critical hyperparameter that determines the step size at each iteration during model training. It controls the speed of gradient descent and affects the performance of the model. However, choosing an optimal learning rate can be challenging. If the learning rate is set too high, the model may learn too quickly, resulting in convergence problems. Conversely, a learning rate that is too low may lead to slow learning, which is ineffective and can result in overfitting or becoming trapped in a local minimum. Therefore, selecting an appropriate learning rate is essential for achieving global minimum and successfully training the model.

##### Max Epoch, Mini Batch Size and Iteration

In neural network learning, an epoch refers to a complete iteration over the entire dataset. In MATLAB, the Max Epoch parameter is used to set the total number of epochs before the network training is stopped. However, when the size of each dataset is large, it may not be possible to process all the data at once due to limited memory resources. In such cases, the data are divided into smaller subsets called batches, with each batch containing a certain number of samples. In addition, the number of samples in each batch is referred to as the batch size. It is important to choose an appropriate batch size as it affects the performance of the neural network during training. Using a large batch size may accelerate the training process, but it can also cause overfitting where the network becomes excessively attuned to the training data, resulting in poor performance on new data. On the other hand, a small batch size can lead to slower convergence, but it also makes the training process more robust and generalizable to new data. Thus, choosing the right batch size is crucial in achieving good performance in neural network training.

The concept of Iteration is closely related to batch size. For instance, if a dataset contains 10 samples and the batch size is set to 2, then it would take 5 iterations to complete one epoch of training. During each iteration, the neural network updates its parameters based on the gradients calculated from the samples in the current batch. The relationship between dataset size, batch size, and iterations can be expressed mathematically, as shown in Equation (1):

(1) Data set size=IterationBatch size(1 Epoch)

#### 2.1.3. Cropping Dental Implant by YOLOv2

**Figure 3:**
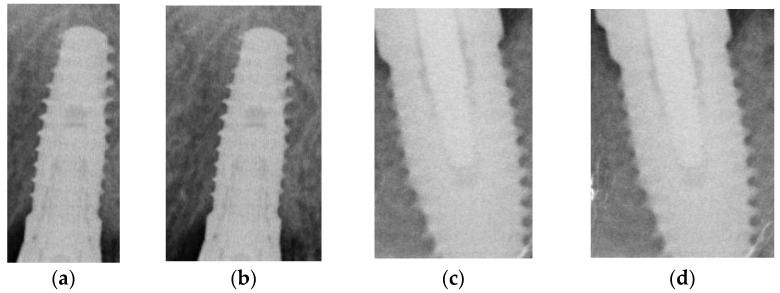
The cropping example with and without extending: (**a**,**c**) the image cropped directly; (**b**,**d**) the image cropped after extending.

The detector after training will return the position of the object, including the dot in the upper left corner of the object and the length and width of the ROI. The key point of this step is to use the returned value to crop the required image. In 2.1.1, it is necessary to preserve the features between the implant and the gingiva to the greatest extent possible during cropping. Therefore, the returned data will add several pixels to the horizontal field as shown in Figure 3.

The detection of damages in implant screw threads is not based on a single implant, but rather on a single side. Thus, after cropping the region of interest (ROI) of the implant thread using YOLOv2, further segmentation is necessary. To simplify the classification of items in the CNN model database and enable the model to focus more on damage identification, the cropped image is segmented into the mesial and distal sides. However, cropping poses a challenge as the thread may not be parallel to the Y-axis of the image. Therefore, linear regression analysis [28] is employed to determine the position of the implant in the image for cropping purposes, as shown in Equation (2):

(2) $$y=\beta_{0}+\beta_{1}x$$

where 0 represents the intercept, and 1 represents the slope. By analyzing the distribution of points on the coordinate axis, a line that represents the overall trend can be obtained. Based on the observation of dental implants in this project, the length of the implant is greater than its width in the photo. Therefore, by placing the implant horizontally on the coordinate axis, a linear equation that passes through the center of the implant can be obtained.

**Figure 4:**
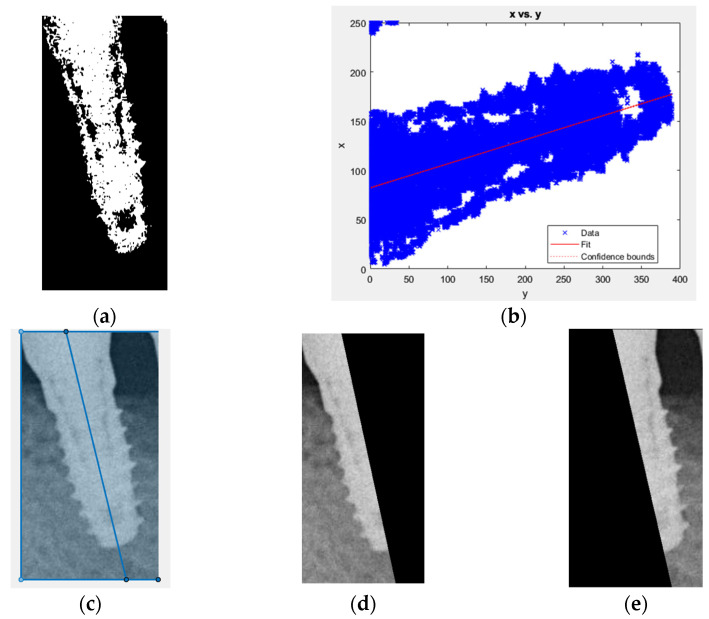
The result of the images: (**a**) image binarization; (**b**) dental implants plot on coordinate axis; (**c**) dental implant midline; (**d**) the left half of the image after cutting; (**e**) the right half of the image after cutting.

The initial step involves the binarization of the image to extract the implant as illustrated in Figure 4a. The following step entails plotting the extracted implant on the XY plane as depicted in Figure 4b. Due to the closely distributed pixels of the implant, the last step involves utilizing linear regression analysis to determine the cutting line via the centerline of the implant. Padding is applied to maintain the symmetry of the cropped image, therefore resulting in two images each containing only half of the screw thread as demonstrated in Figure 4.

### 2.2. Preprocessing

It is crucial to establish a well-characterized database that can effectively aid the CNN model in identifying the presence of peri-implantitis. In order to achieve this, this research categorized the database into two groups: the control group, consisting of implants without signs of peri-implantitis; and the test group, consisting of implants with signs of peri-implantitis. To classify the database, this research consulted and referred to the assessment of three physicians with at least five years of clinical experience on whether the model has detected peri-implantitis. Although the cropped images can be used as a database for the CNN model, the original images still contain significant amounts of noise. This noise hinders the ability of the CNN model to differentiate between damage and health. To enhance the learning ability of the CNN model, it is necessary to remove the noise and improve the features to make the differences between damaged and healthy more distinct. For instance, in the test group’s data, implants with signs of peri-implantitis exhibit obvious black subsidence marks around the alveolar bone on the image, which is not present in the control group’s data. Hence, this research proposed the steps for image enhancement to improve efficacy of the CNN model in detecting peri-implantitis.

The first step is to filter out any unnecessary noise. This involves converting the RGB images to grayscale and using histogram equalization and adaptive histogram equalization to accomplish this. The resulting images are overlaid onto the original images to enhance their boundaries. The second step is to enhance the features by examining the differences in color levels between the implant and gingiva. The research plots the values of each pixel in a 3D space and colors them accordingly on the original image. These steps are then combined to produce a pre-processed image. A CNN model training database is created using these pre-processed images which possess the necessary features and sufficiently high recognition accuracy to enable more effective CNN model training.

#### 2.2.1. Histogram Equalization and Adjust Histogram Equalization

**Figure 5:**
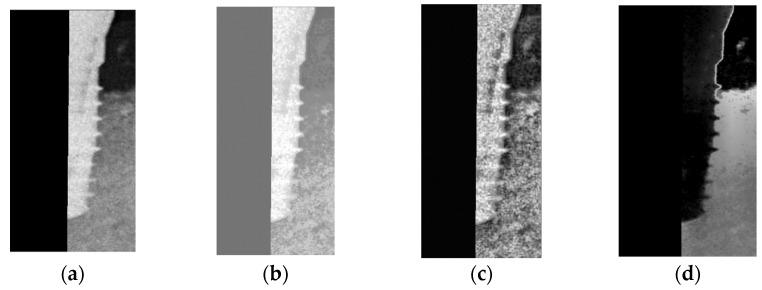
The results of pre-processing: (**a**) original image; (**b**) image after histogram equalization, (**c**) image after adaptive histogram equalization; (**d**) result after subtraction.

**Figure 6:**
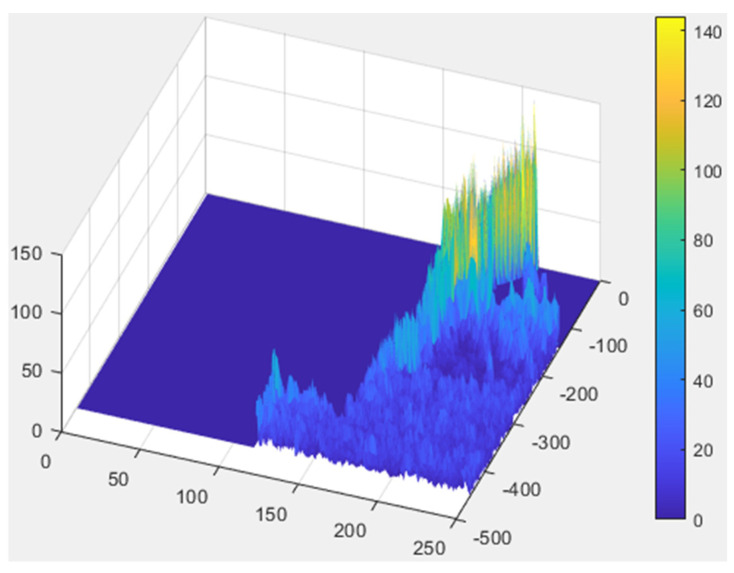
The plot of gradient result in 3D map.

The original image in Figure 5a has a color scale that is too similar between the gingival and screw threads; this makes it difficult to distinguish damaged features due to excessive noise. The main objective of this step is to increase the color scale between the gingival and implant while filtering out the unnecessary noise. Histogram equalization [29] (Figure 5b) and adaptive histogram equalization [30] (Figure 5c) are used to achieve this goal. The result in Figure 5d is obtained by subtracting one image from the other. Then, the norm of the horizontal and vertical gradients of the image is calculated and the results are plotted in 3D as shown in Figure 6, capturing the edge features. Finally, the results are combined with the coloring from the next step to complete the preprocessing. In Equation (3), $p_{x}\left(i\right)$ is the probability value of the occurrence of grayscale values from 0 to 255, $n_{i}$ is the total number of occurrences of grayscale value *i* in the picture, and *n* is the total number of pixels in the image and *L* is 256. Equation (4) presents the cumulative distribution function which calculates the cumulative probability of pixels from 0 to 255 and linearizes the probability of the occurrence of all pixels. Finally, it multiplies 255 by the cumulative distribution function as shown in Equation (5) to scale the cumulative probability of 0 to 1 to 0 to 255.

(3) $$p_{x}\left(i\right)=n_{i}/n\;,\;0\leq i<L$$

(4) $$cdf_{x}\left(i\right)=\overset{i}{\underset{j=0}{\sum }}p_{x}\left(j\right)$$

(5) $$cdf_{x}\left(i\right)\times \left(255-0\right)$$

#### 2.2.2. Image Enhanced with 3D Graphics Technology

**Figure 7:**
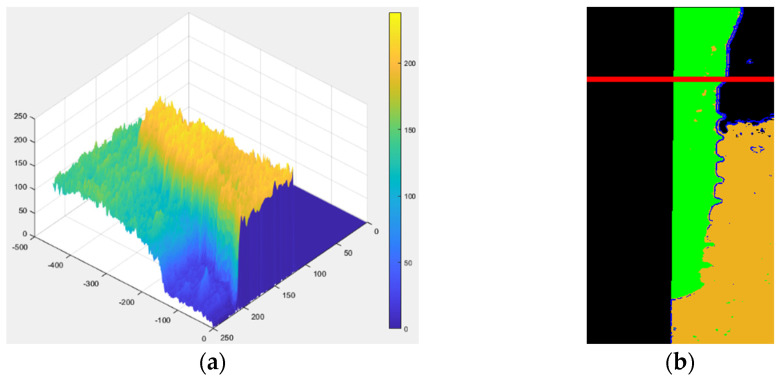
Enhanced schematics through 3D graphics technology: (**a**) the plot of the original image in a 3D map; (**b**) changing the color of the dental implant and gums in the image with added reference lines.

In the previous step, we were able to identify edge features. However, to further emphasize the differences between damaged and healthy, it is necessary to use the distribution of gingiva on the image to enhance the distinction between the two categories of features. The main challenge in this step is distinguishing between the gum and dental implant regions. To address this issue, the correlation between the 3D map output of the previous step and the 3D map of the original image is utilized as shown in Figure 7a. The range value is used to determine whether a pixel is located on the edge or on the flat surface. When a pixel is on the flat surface, the Z-axis position in the 3D map is used as reference to determine whether it belongs to the dental implant or gum. If the Z-axis position is higher than the threshold between the dental implant and gum, the pixel is considered a dental implant and is colored green. If it is lower, it is considered gum and colored orange. A gate value is also used to separate the gum region from the rest of the original image. Pixels below this gate are set to 0 which appears black. Finally, to enhance the discriminability, a red reference line is added at the position of the damaged platform on each image as shown in Figure 7b.

### 2.3. Image Classification

To monitor the learning progress of the model during training, the project divides the training data into an 80% training set and a 20% validation set as listed in Table 5. The validation set is used to observe if the model is overly focused on the training data, leading to incorrect predictions of new data, known as “Overfitting”. Insufficient data is a factor contributing to model overfitting. This project augments the data by horizontally and vertically flipping images, therefore increasing the data volume by a factor of four. To ensure the accuracy of the training process, the number of damaged and healthy data in the training and validation sets must be adjusted to approximately 1:1 to ensure that each category has a consistent distribution of the probability of correct predictions.

#### 2.3.1. CNN Model

**Figure 8:**
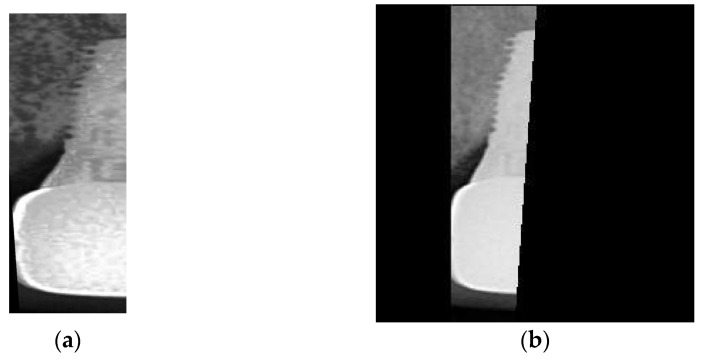
The image used for training the CNN: (**a**) distortion image; (**b**) over padding image.

The hardware setup used to train the CNN image classification model is the same as described in Table 1 in Section 2.1.2. The CNN model is built using the Deep Network Designer app of MATLAB with AlexNet as the base model. However, the input size is different from the original 227 × 227 × 3 and is set to 450 × 450 × 3 to accommodate the elongated shape of dental implants and avoid distortion caused by stretching rectangular images into squares as shown in Figure 8a. This approach also prevents excessive padding resulting from filling the square as shown in Figure 8b. The final architecture of AlexNet is presented in Table 6. To ensure accuracy in the training process, it is necessary to adjust the quantity of damaged and health data in the training and validation sets to approximately 1:1.

#### 2.3.2. Hyperparameter

To train a model effectively, it is crucial to tune the appropriate training parameters according to the data characteristics. The parameters used in the YOLOv2 model trained in Section 2.1.2 differ from those used in the AlexNet model in this step. In this section, we will provide details about the Initial Learning Rate, Mini Batch Size, Max Epoch, and Dropout Factor. Moreover, the parameters used to train AlexNet are presented in Table 7.

##### Learning Rate Dropout

Machine learning models must be generalized to all types of data within their respective domains to make accurate predictions. Overfitting happens when a model becomes too closely fitted to the training dataset and fails to generalize. Preventing overfitting is crucial for successful model training. Dropout is a regularization technique utilized to address overfitting. It involves assigning a probability of dropping out hidden layer neurons during each iteration or epoch of training. The dropped-out neurons do not transmit any information.

## 3. Results

This chapter is divided into two sections: the first focuses on the training process and results of the YOLOv2 object detection model; while the second covers the CNN image classification model. Both models will be compared with those proposed in other papers, and the precision and accuracy achieved by this project will be assessed using the confusion matrix.

### 3.1. YOLOv2 Object Detector

**Figure 9:**
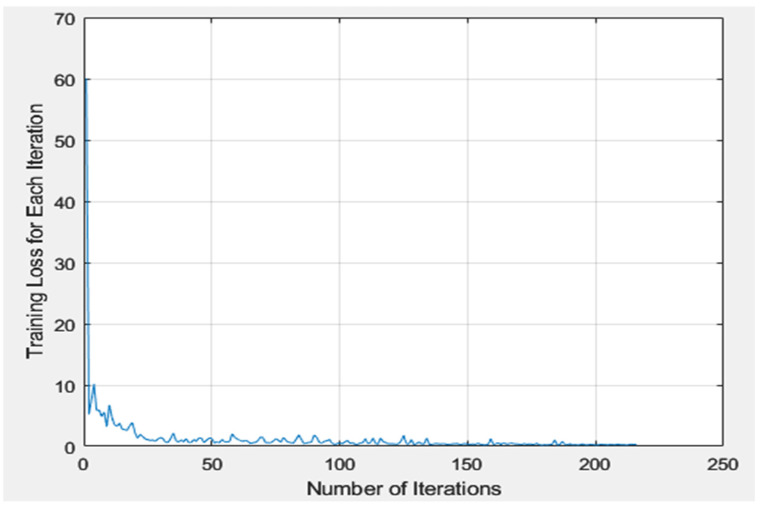
The training process for the YOLOv2 loss function. The blue line represents the change in the loss function over the number of iterations.

Table 8 presents a comprehensive overview of the YOLOv2 training process employed in this study. In this study, an unvalidated model was utilized to detect dental implants due to the fact that the YOLOv2 training function in MATLAB does not support validation. The results of the detection process are detailed in the confusion matrix provided in Table 9. Moreover, Figure 9 depicts the training process for the YOLOv2 loss function. To evaluate the accuracy of the CNN model, the validation set was utilized as the input for the network in this study. The accuracy of the AlexNet model was evaluated by comparing its predictions with the correct answers obtained from the images.

**Figure 10:**
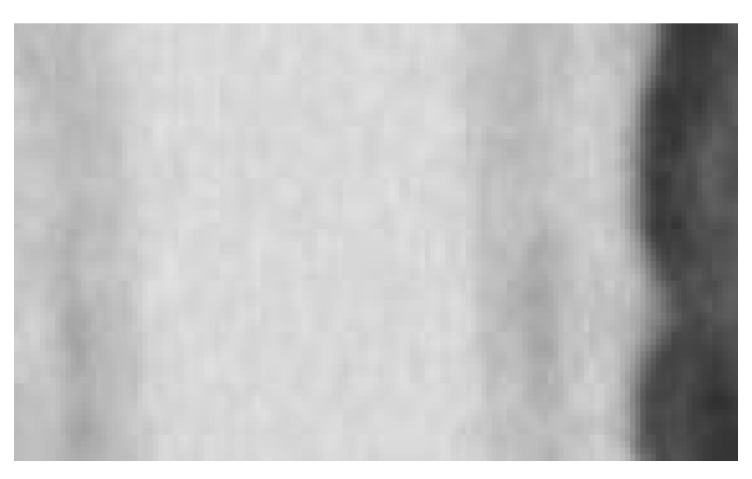
The image of detecting an incomplete implant.

The appropriate selection of hyperparameters is crucial for the success of any machine learning algorithm. In this study, the hyperparameters of YOLOv2 were carefully selected based on the data characteristics. Zero (0) indicates that the YOLOv2 model correctly predicted 287 implants across all test cases, achieving a recall of 90.5%. Additionally, it correctly predicted 89 cases of normal teeth, resulting in a true negative rate of 78%. The accuracy rate of the model in this study is 89.3%. In addition, the model is 95% accurate. The YOLOv2 model displays lower propensity for erroneously detecting healthy teeth, but another issue was encountered during testing. As depicted in Figure 10, the system tends to repeatedly detect incomplete implants in the same tooth leading to high false negative values. In contrast to literature [31,32] that employ positioning and identification technology, the proposed technology in this study integrates automatic image cropping, resulting in a difference in the accuracy of less than 2%. This proposal has attained high precision and accuracy in dental implant detection and image classification. In general, these outcomes suggest that our proposed models exhibit a potential for clinical applications and could serve as a valuable tool for dental implant planning.

### 3.2. CNN AlexNet Image Classification

**Figure 11:**
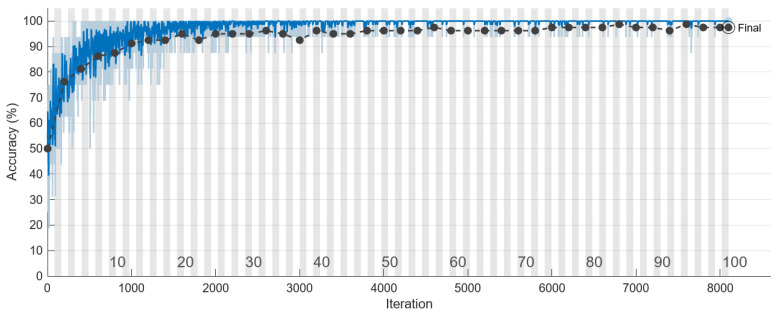
The accuracy of AlexNet model in the validation set (black line) and training set (blue line) during training process.

**Figure 12:**
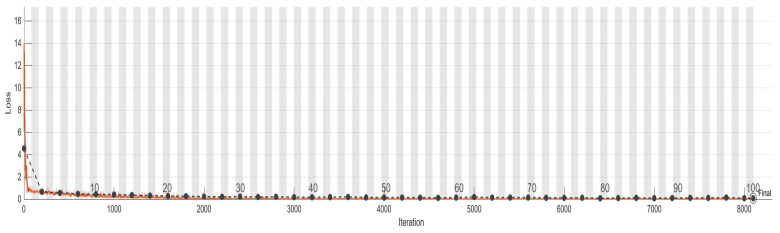
The accuracy of AlexNet model in the validation set (black line) and the training set (orange line) during loss training process.

To monitor the training progress of the model, a validation set was employed in this project. The training process is presented in Table 10, while Figure 11 and Figure 12 display the accuracy and loss of training AlexNet, respectively. The black line in both figures represents the validation.

Based on the data presented in Table 11, it is evident that the use of distorted images, which were not adjusted for relative size, as the training database led to a very high loss of the validation set and an accuracy of less than 50%. These findings suggest that the database may have some fatal flaws such as indistinct features resulting from image stretching or excessive noise in the image. The second column of Table 11 presents the results of training using histogram equalization, the overlaid original image, and adaptive histogram equalization. As a result of correcting image size and enhancing features, the loss decreased significantly, and the accuracy increased to 81.43%. Nonetheless, these results were still below the standards set by this project. Furthermore, during the training process, the loss rebounded after reaching 0.5, indicating the need for further image pre-processing. The third column of Table 11 shows the result of this project which significantly enhances the features of an image by coloring different regions and adding damage reference lines. This enhancement has helped the model to perform better in image classification, achieving a validation set accuracy of 97.5%, and the loss has also dropped below the threshold of 0.5 to 0.08.

The evaluation of prediction results in terms of accuracy and precision was performed by comparing the predicted outcomes with the ground truth using a confusion matrix based on the test set, as presented in Table 12. Initially, the AlexNet model employed original images during the training process, and after continuous adjustments, the accuracy increased from 60% to 80%. However, it reached a bottleneck. Further improvement was achieved with the use of preprocessing. The final training outcomes are depicted in Table 12. Nevertheless, some images in the test data have unclear boundaries such as those without obvious screw threads or gums having similar grayscale values to screw threads which may also lead to misjudgment by the human eye. Consequently, there were approximately 10% errors in the final testing. It is evident that the CNN model performed remarkably well on the test set; it accurately predicted 107 out of all 117 samples, with damages accounting for 91.4% of the total damaged test data; similarly, it accurately predicted 92 out of all 103 healthy samples, accounting for 89.3% of the total healthy test data. The accuracy rate was 90.4% and the precision rate was 90.7%.

The direct classification of dental implant damages has not yet been addressed in the current state of the art. The paper that is closest in technique is [33] which focuses solely on determining the fit between two sides of a dental implant rather than directly detecting damages on one side of the implant using the method employed in this project. Table 13 presents a comparison with this technology, revealing that the image enhancement technology utilized in this study contributes to the final recognition result of the CNN, with an accuracy rate increasing to 90.4%. This marks significant progress, as this research currently represents the highest performance in detecting implant damages in teeth.

## 4. Discussion

The YOLOv2 model achieved an 89.3% accuracy rate in detecting the position of dental implants, surpassing the performance of existing methods [34]. The multiple identification process revealed that the system may repeatedly detect the same tooth when there are incomplete implants, leading to high false negative values. In addition to the positioning and recognition technology used in [31,32], this study introduced automatic image cropping, resulting in less than a 2% accuracy difference. This is a promising direction. Another study [35] used YOLOv3 to identify dental implants, with TP ratios and APs ranging from 0.50 to 0.82 and 0.51 to 0.85 for each implant system, respectively. The resulting mAP and mIoU of the model were 0.71 and 0.72, respectively. This is with a small amount of training data used, which may have compromised the accuracy of the model. For the AlexNet data used in this study, grayscale images were initially used for training, resulting in lower accuracy rates. When distorted images were used, the accuracy rate was even lower. Therefore, this research strengthens the high-precision image preprocessing process, improves the accuracy of the model to detect damages to 90.4%, and innovates and breaks through the latest similar related research.

The most related investigation [33] utilized Faster R-CNN to identify marginal bone loss surrounding implants (the κ value for the bone loss site was 0.547, while the κ value for the bone loss implant was 0.568) and compared the judgments of the AI to those of MD students and resident dentists on the same data. The results showed significant differences in the judgments of human observers. Therefore, training a consistent and accurate model can greatly facilitate healthcare by providing real-time treatment. However, the model is limited in its ability to detect finer levels of bone loss or the number of threads affected. Future research could address this limitation by exploring the use of additional imaging techniques or developing more sophisticated algorithms to detect these features, reducing misjudgment, and avoiding medical disputes.

## 5. Conclusions

The YOLOv2 model achieved an accuracy rate of 89.3% in capturing the implant position, while the AlexNet damage detection model achieved an accuracy rate of 90.4%. Moving forward, this research will continue to optimize the model and investigate better methods to improve accuracy rates. In terms of capturing dental implants, this study improves the cropping method by cutting through the interdental space to avoid capturing teeth outside the target area. In addition, this study obtained different grayscale ranges and blurred glue and line edges in the image. This automatic image preprocessing method greatly improves on the current manual preprocessing process. This automated approach not only improves efficiency and consistency but also reduces manual operation errors. Moreover, it is advisable to investigate the potential of incorporating advanced imaging techniques or developing more sophisticated algorithms that can accurately detect even subtle levels of bone loss or the number of affected threads. Moreover, creating a user interface can improve user satisfaction and increase the ease of use and efficiency of the system, leading to improved work efficiency and product quality.

## Figures and Tables

**Table 1:** Experimental environment specifications.

**Hardware Platform**	**Version**
CPU	Intel i5-12400
GPU	GeForce RTX 3080
DRAM	DDR4 3200 32 GB
**Software Platform**	**Version**
MATLAB	R2022b

**Table 2:** The Layers of YOLOv2 model.

	Type	Activations
1	Image Input	416 × 416 × 3
2	2-D Convolution	416 × 416 × 16
3	Batch Normalization	416 × 416 × 16
4	Leaky ReLU	416 × 416 × 16
5	2-D Max Pooling	208 × 208 × 16
6	2-D Convolution	208 × 208 × 32
7	Batch Normalization	208 × 208 × 32
8	Leaky ReLU	208 × 208 × 32
9	2-D Max Pooling	104 × 104 × 32
10	2-D Convolution	104 × 104 × 64
11	Batch Normalization	104 × 104 × 64
12	Leaky ReLU	104 × 104 × 64
13	2-D Max Pooling	52 × 52 × 64
14	2-D Convolution	52 × 52 × 128
15	Batch Normalization	52 × 52 × 128
16	Leaky ReLU	52 × 52 × 128
17	2-D Max Pooling	26 × 26 × 128
18	2-D Convolution	26 × 26 × 256
19	Batch Normalization	26 × 26 × 256
20	Leaky ReLU	26 × 26 × 256
21	2-D Max Pooling	13 × 13 × 256
22	2-D Convolution	13 × 13 × 512
23	Batch Normalization	13 × 13 × 512
24	Leaky ReLU	13 × 13 × 512
25	2-D Max Pooling	13 × 13 × 512
26	2-D Convolution	13 × 13 × 1024
27	Batch Normalization	13 × 13 × 1024
28	Leaky ReLU	13 × 13 × 1024
29	2-D Convolution	13×13×512
30	Batch Normalization	13×13×512
31	Leaky ReLU	13 × 13 × 512
32	2-D Convolution	13 × 13 × 30
33	Transform Layer	13 × 13 × 30
34	Output	13 × 13 × 30

**Table 3:** Hyperparameters for YOLOv2 training.

Hyperparameters	Value
Optimizer	sgdm
Initial Learning Rate	0.001
Max Epoch	24
Mini Batch Size	16

**Table 4:** The distribution of data in the original periapical image obtained from clinical sources.

The Number of Original Periapical Images				
	Training	Test	The Others	Total
Quantity	147	46	263	456

**Table 5:** Image classification of the periapical image before and after preprocessing.

The Number of Periapical Images before and after Preprocess		
Before	Training Set	Validation Set
Healthy	162	40
Damaged	164	40
Total	326	80
After	Training Set	Validation Set
Healthy	648 (Augmented)	40
Damaged	656 (Augmented)	40
Total	1304	80

**Table 6:** The model architecture of AlexNet.

	Type	Activations
1	Image Input	450 × 250 × 3
2	2-D Convolution	110 × 60 × 96
3	ReLU	110 × 60 × 96
4	Cross Channel Normalization	110 × 60 × 96
5	2-D Max Pooling	54 × 29 × 96
6	2-D Grouped Convolution	54 × 29 × 256
7	ReLU	54 × 29 × 256
8	Cross Channel Normalization	54 × 29 × 256
9	2-D Max Pooling	26 × 14 × 256
10	2-D Convolution	26 × 14 × 384
11	ReLU	26 × 14 × 384
12	2-D Grouped Convolution	26 × 14 × 384
13	ReLU	26 × 14 × 384
14	2-D Grouped Convolution	26 × 14 × 256
15	ReLU	26 × 14 × 256
16	2-D Max Pooling	12 × 6 × 256
17	Fully Connected	1 × 1 × 1152
18	ReLU	1 × 1 × 1152
19	Dropout	1 × 1 × 1152
20	Fully Connected	1 × 1 × 144
21	ReLU	1 × 1 × 144
22	Dropout	1 × 1 × 144
23	Fully Connected	1 × 1 × 2
24	Softmax	1 × 1 × 2
25	Classification Output	1 × 1 × 2

**Table 7:** Hyperparameters in AlexNet model.

Hyperparameters	Value
Optimizer	Sgdm
Initial Learning Rate	0.00006
Max Epoch	50
Mini Batch Size	16
LearnRateDropFactor	0.75
LearnRateDropPeriod	30

**Table 8:** Training process for YOLOv2.

Epoch	Iteration	Time Elapsed	Mini-Batch RMSE	Mini-Batch Loss
1	1	00:03	7.75	60.0
6	50	00:27	1.13	1.3
12	100	00:51	0.77	0.6
17	150	01:14	0.56	0.3
23	200	01:37	0.44	0.2
24	216	01:43	0.53	0.3

**Table 9:** The confusion matrix of YOLOv2 test.

Target Class				
Category		Implant	Tooth	Subtotal
Output Class	Implant	287 (68.2%)	15 (3.6%)	95%
	Tooth	30 (7.1%)	89 (21.1%)	74.8%
	Subtotal	90.5%	78%	89.3%

**Table 10:** The detailed process of AlexNet training.

Epoch	Iteration	Mini-Batch Accuracy	Validation Accuracy	Mini-Batch Loss	Validation Loss
1	1	56.25	50.00	8.3222	3.1206
10	810	81.25	87.50	0.2822	0.2692
20	1620	100.00	95.00	0.0358	0.1354
30	2430	100.00	96.25	0.0376	0.1144
40	3240	100.00	96.25	0.0177	0.0951
50	4050	100.00	96.25	0.0126	0.0676
60	4860	100.00	97.50	0.0070	0.0989
70	5670	100.00	97.50	0.0109	0.0740
80	6480	100.00	97.50	0.0051	0.0672
90	7290	100.00	97.50	0.0035	0.0551
100	8100	100.00	97.50	0.0055	0.0766

**Table 11:** Comparison of the datasets used in various stages and the validation results.

	Original Images	Adaptive Histogram Equalization	Adaptive Histogram Equalization + Damage Reference Lines
Validation Accuracy	48.64	81.43	97.5
Validation Loss	2.21	0.54	0.08
Net	AlexNet	AlexNet	AlexNet
Image	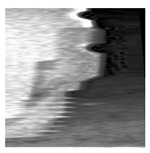	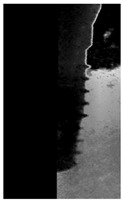	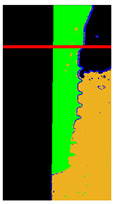

**Table 12:** The confusion matrix of AlexNet test.

		Target Class		
Category		Damaged	Healthy	Subtotal
Output Class	Damaged	107 (48.6%)	11 (5%)	90.7%
	Healthy	10 (4.5%)	92 (41.8%)	90.2%
	Subtotal	91.4%	89.3%	90.4%

**Table 13:** The comparison table between the prior art and this study.

	This Work	Method in [33]
Method	CNN	Faster R-CNN
Accuracy	90.4%	81%

## Data Availability

Not applicable.
